# Type 2 Diabetes Elicits Lower Nitric Oxide, Bradykinin Concentration and Kallikrein Activity Together with Higher DesArg^9^-BK and Reduced Post-Exercise Hypotension Compared to Non-Diabetic Condition

**DOI:** 10.1371/journal.pone.0080348

**Published:** 2013-11-12

**Authors:** Herbert Gustavo Simões, Ricardo Yukio Asano, Marcelo Magalhães Sales, Rodrigo Alberto Vieira Browne, Gisela Arsa, Daisy Motta-Santos, Guilherme Morais Puga, Laila Cândida de Jesus Lima, Carmen Sílvia Grubert Campbell, Octavio Luiz Franco

**Affiliations:** 1 Programa de Pós-Graduação em Educação Física, Universidade Católica de Brasília, Brasília, DF, Brasil; 2 Programa de Pós-Graduação em Educação Física, Universidade Federal de Mato Grosso, Cuiabá, Mato Grosso, Brasil; 3 Departamento de Fisiologia and Biofísica, Instituto de Ciências Biológicas, Universidade Federal de Minas Gerais, Belo Horizonte, Minas Gerais, Brasil; 4 Departamento de Educação Física, UNESP, Rio Claro, SP, Brasil; 5 Departamento de Educação Física, UFU, Uberlândia, Minas Gerais, Brasil; 6 Centro de Análises Proteômicas e Bioquímicas, Universidade Católica de Brasília, Brasília, DF, Brasil; 7 Programa de Pós-Graduação em Imunologia e DIP/ Genética e Biotecnologia, Universidade Federal de Juiz de Fora, Minas Gerais, Brasil; Albany Medical College, United States of America

## Abstract

This study compared the plasma kallikrein activity (PKA), bradykinin concentration (BK), DesArg^9^-BK production, nitric oxide release (NO) and blood pressure (BP) response after moderate-intensity aerobic exercise performed by individuals with and without type 2 diabetes. Ten subjects with type 2 diabetes (T2D) and 10 without type 2 diabetes (ND) underwent three sessions: 1) maximal incremental test on cycle ergometer to determine lactate threshold (LT); 2) 20-min of constant-load exercise on cycle ergometer, at 90% LT and; 3) control session. BP and oxygen uptake were measured at rest and at 15, 30 and 45 min post-exercise. Venous blood samples were collected at 15 and 45 minutes of the recovery period for further analysis of PKA, BK and DesArg^9^-BK. Nitrite plus nitrate (NOx) was analyzed at 15 minutes post exercise. The ND group presented post-exercise hypotension (PEH) of systolic blood pressure and mean arterial pressure on the 90% LT session but T2D group did not. Plasma NOx increased ~24.4% for ND and ~13.8% for T2D group 15min after the exercise session. Additionally, only ND individuals showed increases in PKA and BK in response to exercise and only T2D group showed increased DesArg^9^-BK production. It was concluded that T2D individuals presented lower PKA, BK and NOx release as well as higher DesArg^9^-BK production and reduced PEH in relation to ND participants after a single exercise session.

## Introduction

Type 2 diabetes (T2D) is associated with endothelial dysfunction, disautonomy and arterial hypertension [1–3]. Endothelial dysfunction, often observed in subjects with T2D, may affect the release of vasodilator substances such as prostaglandins, kallikrein (KLK), bradykinin (BK) and nitric oxide (NO) [4,5] and thus might impair physical exercise induced vasodilation. 

It has been shown that a single session of exercise can promote significant reductions in blood pressure (BP) that last for minutes or hours after exercise compared to pre-exercise values [6–8]. This phenomenon is known as post-exercise hypotension (PEH) and may be associated with the activity of the kallikrein-kinin system and NO release, among other factors. Physical exercise has been pointed out as an effective non-pharmacological intervention for hypertension control [9]. The prescription of exercise intensities based on lactate threshold (LT) has been suggested for individuals with T2D [6–8,10,11] since LT represents an intensity of low cardiovascular and metabolic stress, and therefore may be considered safe [12–14]. 

Although studies have observed PEH in individuals with T2D [6–8,15], the molecular mechanisms underlying its occurrence have not been fully elucidated. Moreover, in spite of previous studies showing that individuals with T2D exhibit PEH when exercising at intensities around LT [7,8,15], Motta et al. [6] found that subjects with T2D exhibit lower plasma kallikrein activity (PKA) compared to healthy individuals, suggesting that lower NO release and reduced endothelium-dependent vasodilation would be occurring in response to exercise for this population. However, no investigations were made on the integrated responses of PKA, BK, DesArg^9^-BK and NO and its association to the occurrence of PEH in individuals with or without T2D.

So, the present study investigated and compared the responses of PKA, BK, DesArg^9^-BK, NOx and BP after a single session of moderate-intensity aerobic exercise in T2D and ND individuals. We hypothesized that, in comparison to non-diabetic individuals, those with T2D would present attenuated PEH as a consequence of reduced activity and release of vasodilator substances that compose the kallikrein-kinin system, such as PKA, BK and its metabolite (DesArg^9^-BK) as well as NO. These different patterns of response in T2D would be related to the endothelial dysfunction commonly observed in this population [16–18].

## Materials and Methods

The protocols used in the present study were approved by the Ethics Committee on Human Research (SES/DF n° 087/2007, Brazil) and are in accordance with the Declaration of Helsinki (1964). Prior to participation, all subjects received a full explanation about the purposes and procedures of the study and gave a written informed consent.

### Subjects

Ten individuals with and ten individuals without T2D, all sedentary, participated in the study. Their characteristics are presented in table 1. All individuals with T2D were taking oral hypoglycemiants such as *Sulfonylureas, Metformin, Metformin+Glibenclamide, Glimepiride, Pioglitazone Chloridrate* and none of them was under insulin treatment. Five of them were taking anti-hypertensive medication such as calcium channel antagonists and diuretics. All medication was washed-out for 48h prior to the initial screening visit and the three subsequent experimental sessions. The individuals were also asked to avoid physical exercises and alcoholic or caffeinated drinks for 24h prior to each visit to the lab. The exclusion criteria included: history of cerebral stroke or acute myocardial infarction, severe secondary complications (such as blindness or wounded diabetic foot), physical or cardiovascular problems that could impair the accomplishment of exercise, target-organ damage, current or previous tobacco use, pre-menopause women and age under 40 or over 60 years. 

**Table 1:** General characteristics of T2D and ND groups.

	**T2D (n=10)**	**ND (n=10)**
**Age (years)**	53.2±6.2	48.0±5.0
**Gender (men/women)**	8/2	9/1
**Time of T2D diagnosis (years)**	6.0±1.1	-
**Body Weight (kg)**	90.0±6.1	83.3±3.5
**Height (m)**	1.71±0.02	1.72±0.02
**BMI (kg.m^-2^)**	30.7±1.8	28.0±1.2
**HbA_1c_ (%)**	6.9±0.3*	5.2±0.4
**Blood Glucose (mg.dL^-1^)**	167.0±11.2*	91.8±2.6
**VO_2_peak (mL.kg^-1^.min^-1^)**	20.1±1.7*	28.1±2.4
**Peak power output (W)**	108.3±9.1	138.2±11.1
**Lactate threshold (W**)	68.1±7.1	79.1±7.1

Data expressed as mean and (±) standard deviation. T2D= type 2 diabetic group; ND= non-diabetic group; BMI= body mass index; HbA_1c_= glycated hemoglobin estimated as proposed by Rohlfing et al. [39]; VO_2_peak= peak of oxygen consumption; *= p<0.05 in relation to ND group.

### Screening visit

After a 12-hr fasting, body weight (kg), height (cm) and abdominal circumference (cm) were measured. A capillary blood sample was collected for the determination of fasting blood glucose concentration (Accu-check Advantage, Roche, German) and for estimation of glycated hemoglobin, as previously suggested [19] (Table 1). 

### Sessions

All experimental sessions started two hours after the ingestion of a standardized breakfast. It was considered to have a moderate glycemic index (GI = 73.9) [20] and consisted of 315.9 kcal, as follows: 53 g (67.1% - 212 kcal) of carbohydrate; 4.6 g (5.8% - 18.3 kcal) of protein and 9.5 g (27.1% - 85.6 kcal) of fat. 

The following sessions were performed on distinct days, separated by at least 72h and always beginning at 8 am: 1) Incremental Exercise Test (IET); 2) moderate aerobic exercise (EX) (90% of lactate threshold); 3) control session (CON). The EX and CON sessions were performed in randomized order. All the experimental sessions comprised the following periods: 

### Pre and post exercise procedures

 Prior to EX and CON sessions, the participants remained at seated rest and the blood pressure (BP) and heart rate (HR) were measured every 5 min for 20 min (BP 3AC1-1, Microlife AG, Widnau, Switzerland; Polar® S810i, Polar Electro Oy, Finland). At the same time points, capillary blood samples (25μL) were collected from the ear lobe for blood lactate (bLac) and blood glucose (bGluc) determination (YSI 2700, YSI Inc, Yellow Springs, OH, USA). During the post-exercise period, the volunteers remained seated and BP was measured every 15 min for 45 min. Furthermore, at the end of the 20-min pre-session resting period, as well as at 15 (rec15) and 45 min (rec45) of post-exercise or control period, a venous blood sample was drawn for the determination of BK concentration, as well as of plasma kallikrein activity and DesArg^9^-BK level. Additionally, NOx concentration was analyzed at resting and at rec15.

### Incremental Exercise Test

The IET was performed on an electromagnetic cycle ergometer (Lode Excalibur Sport - Netherlands) with an initial workload of 15 watts followed by 15 watts increments at each 3 min stage until volitional exhaustion, and a constant pace of 60 revolutions per minute as previously suggested [10,13]. The ECG trace was monitored for each individual during the whole test in order to identify any possible abnormality during exercise. At the end of the pre-exercise resting period, as well as at the last 20s of each incremental stage, a 25 μL blood sample was collected from the ear lobe through calibrated heparinized glass capillaries, and deposited in microtubes (*Eppendorf*) containing 50 μL of sodium fluoride (NaF) 1% for later analyses of bLac and bGluc using an electroenzimatic method (Yellow Springs 2.700 STAT, OH, USA).

Expired gases were also measured during the IET through a gas analyzer (Metalyzer 3B, Cortex Biophysik, Leipzig, Germany) previously calibrated with a 3-L syringe (flow calibration) and a standard mixed gas containing 4.9% CO_2_ and 17% O_2_ (gas calibration).

### Lactate Threshold determination, Ventilatory Analysis and *V*O_2_ peak measurement

The LT was visually identified during the IET at the inflection point of the bLac versus workload curve, by 2 or more experienced researchers [14,21]. Expired gas analyses were performed breath by breath over the test (Metalyzer 3B, Cortex Biophysik, Leipzig, Germany) and VO_2_peak was considered as the highest oxygen consumption attained at the moment of exhaustion during the IET [22].

### Moderate aerobic exercise and control session

The EX session consisted of a 20 min cycle ergometer exercise (Lode Excalibur Sport, Netherlands) at a constant load corresponding to 90% of LT. During the exercise, BP, HR, capillary blood sampling and rate of perceived exertion (RPE) were obtained at the 10^th^ and 20^th^ min. Ventilatory variables were also registered during the entire session. During the CON session, participants remained at seated rest for 20 min with no exercise performance. However, the same procedures of data collection described for EX session were carried out.

### Venous Blood Sampling

Blood samples were collected for NOx, BK, DesArg^9^-BK and PKA analysis in 4 mL Vacutainer® tubes (BD, New Jersey, USA) containing citrate solution at 3.2 % or EDTA. The samples were centrifuged at room temperature for 15 min at 1.500 rpm and 1 mL aliquots of plasma were separated in 1.5mL microtubes and immediately frozen at -80°C until the moment of analysis. 

### Plasma kallikrein activity (PKA)

PKA was determined by spectrofluorometry (HitachiF 2500, Tokyo, Japan), using benzyloxycarbonyl-phenylalanine-arginine-4-amino-7-methylcoumarin (Z-Phe-Arg-AMC; Calbiochem, Merck, Darmstadt, Germany) as substrate. Substrate specificity was determined by specific inhibition with plasma kallikrein serine proteinase inhibitor (PKSI). The reaction was initiated by incubating 5µL of plasma with 2mL of 50mM Tris buffer (pH 7.4), containing 100 mM of NaCl. After a 3 min pre-incubation period, the substrate was added to the cuvette and the reaction was continuously monitored for 300s at 380nm excitation and 460nm emission in a cell compartment set at 37°C. Following this, 5µL of 10-mM PKSI was added to the solution and the reaction was monitored for additional 100s. PKA, expressed as fluorescence arbitrary units per minute (FAU.min^−1^), was calculated as the rate of substrate hydrolysis measured without PKSI minus the rate of substrate hydrolysis measured with PKSI [6,23–26].

### Plasma levels of bradykinin (BK)

BK extraction was carried out using C18 Sep-Pak columns previously activated with 90% Acetonitrile (2 mL), water (5 mL), 5% acetonitrile in 1% phosphoric acid (5 mL). Subsequently to the activation, the samples were applied in the column, washed with 5% acetonitrile in 1% phosphoric acid and eluted at 35% acetonitrile in 1% phosphoric acid. The eluates were lyophilized, dissolved again in 500uL of mobile phase A (5% acetonitrile in 0.1% orthophosphoric acid) and filtered through a 0.22µm membrane for analysis by High Performance Liquid Chromatography (HPLC). The peptides were then separated on a reversed phase column (Aquapore 300 ODS; 250 x 4.6 mm), using isocratic gradient for 5 min followed by 20 min of linear gradient from 5% to 35% mobile phase B (95% Acetonitrile in 0.1% H3PO4), under a flow of 1.5 mL/min for a period of 40 min. BK was identified by comparing the retention time with the standard kinin. All samples were tested in duplicate and the average values were considered.

### Quantification of DesArg^9^-BK

 The residues of the evaporated ethanolic extracts were resuspended in 50 mM Tris^.^HCl buffer, pH 7.4, containing 100 mM NaCl and 0.05% Tween 20. After resuspension, DesArg^9^-BK was quantified by specific chemiluminescent enzyme immunoassays, as previously described [27].

### NO concentrations

To determine NO concentrations, nitrite plus nitrate (NOx) tests were performed in plasma. Plasma samples were ultrafiltrated (Microcontroller Centrifugal Filter Units, 10 kDa, Millipore, Bedford, MA, USA). NOx concentration was determined using a commercial kit (Cayman Chemical, Ann Arbor, MI, USA). The amount of NO was determined from the enzymatic conversion of nitrate to nitrite by nitrate reductase. After conversion, the spectrophotometric measurement of nitrite plus nitrate was conducted by Griess reaction. The resulting compound absorbed light at 540-550 nm [28].

### Statistical Analysis

Data are presented as mean (±) standard deviation or standard error of the mean (SEM), as indicated for each analysis. After assessing normality of the data through the Shapiro-Wilk test, between- and within-groups comparisons were performed by using Split-Plot ANOVA (Mixed ANOVA). When any of the dependent variables did not show sphericity through the Mauchly’s test, the epsilon of Greenhouse-Geisser was used to analyze the F statistic. In addition, Student t-test for independent samples was used for comparing the characteristics of the individuals in the different groups. The level of significance was set at 5% (p≤0.05) and all analysis were carried out using the Statistical Package for the Social Sciences (SPSS) 15.0.

## Results

Table 1 shows the biochemical (bLAC and bGluc), hemodynamic (HR and BP) and respiratory (VO_2_ and VCO_2_) characteristics of the sample. The T2D group showed significantly (p<0.05) higher estimated glycated hemoglobin and blood glucose, and lower VO_2_peak compared to the ND group. Table 2 presents the variables obtained during moderate exercise (90% LT) for T2D and ND groups.

**Table 2:** Workload, biochemical, hemodynamic and respiratory variables during the exercise performed at 90% of lactate threshold for T2D and ND groups.

	**T2D (n=10)**	**ND (n=10)**
**Power Output (W)**	60.2±6.3	70.1±7.2
**VO_2_ (mL.kg.^-1^min^-1^)**	14.2±1.2	15.1±1.2
**HR (bpm)**	115.2±4.1	124.2±4.1
**Blood Lactate (mM)**	3.2±0.9	3.7±0.4
**Blood Glucose (mg.dL^-1^)**	159.2 ±16.3*	60.1±3.2

Data expressed as mean and (±) standard error of the mean. T2D= type 2 diabetic group; ND= non-diabetic group; VO_2_= oxygen consumption; HR= heart rate; *= p<0.05 in relation to ND group.

**Figure 1:**
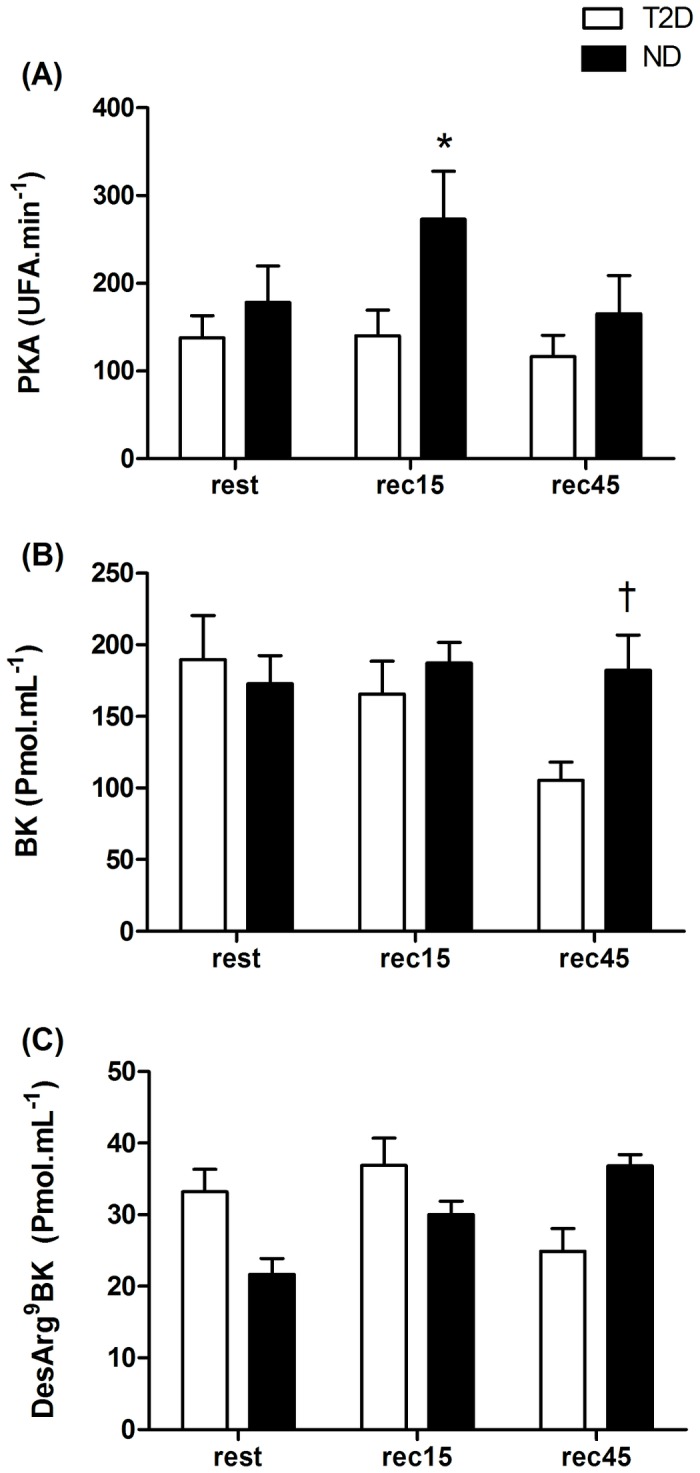
Plasma kallikrein activity – PKA (A), Bradykinin – BK (B) and DesArg^9^-BK (C) at rest and post-exercise recovery period (rec15 and rec45 min) for type 2 diabetic (T2D) and non-diabetic (ND) groups. *= p<0.05 in relation to T2D at rec15; †= p<0.05 in relation to T2D at rec45.

The ND group showed higher PKA (p<0.05) when compared to T2D group at 15 min of post-exercise recovery (rec15). Similarly, BK was higher (p<0.05) for ND group when compared to T2D individuals at 45 min of post-exercise recovery (rec45). In addition, DesArg^9^-BK was higher for T2D individuals when compared to ND group at rec45 (Figure 1).

On sessions EX and CON, the ND group showed higher NOx values at rest as well as after exercise (rec15) compared with T2D group, and for both groups NOx values at rec15 were higher than at the resting period (p<0.05) (Table 3).

**Table 3:** Concentration of nitrite plus nitrate (NOx) (µmol) at pre-exercise resting and post-exercise recovery (rec15, 15^th^ min of recovery) in individuals with and without type 2 diabetes on the moderate aerobic exercise (EX) and control session (CON).

**Session**	**Group**	**Resting**	**Post-exercise (rec15)**
		**(µmol)**	**(µmol)**
**EX**	**T2D**	28.1±0.9	32.0±2.0*
	**ND**	33.6±0.6†	41.8±1.4*†
**CON**	**T2D**	29.0±0.7	26.2±0.5
	**ND**	34.2±0.8†	32.1±1.2†

Data expressed as mean and (±) standard error of the mean. T2D= type 2 diabetic group; ND= non-diabetic group; *= p<0.05 in relation to resting values within group; †= p<0.05 in relation to T2D group.

**Figure 2:**
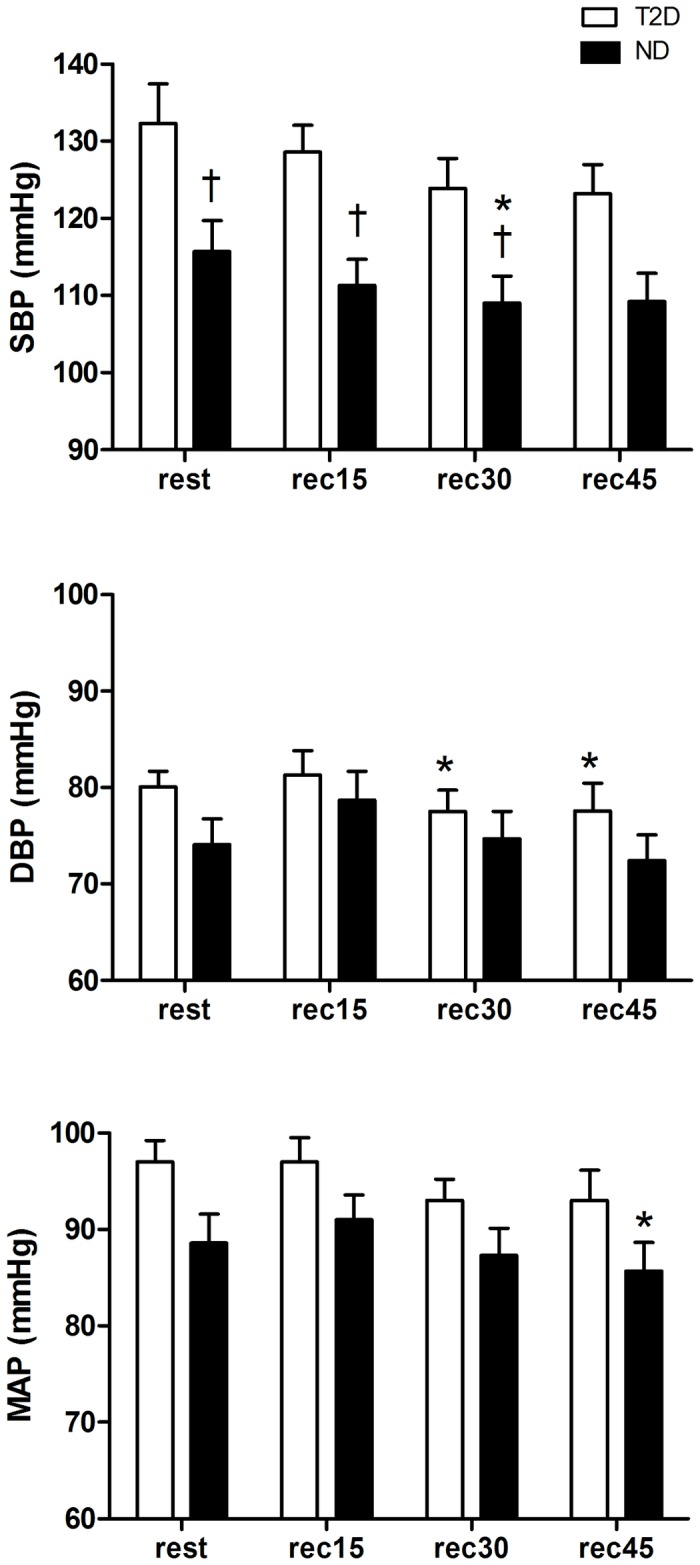
Systolic blood pressure (SBP), diastolic blood pressure (DBP) and mean arterial pressure (MAP) at rest and post-exercise recovery period (rec15, rec30 and rec45 min) for type 2 diabetic (T2D) and non-diabetic (ND) groups. *= p≤0.05 in relation to rest; †= p≤0.05 in relation to T2D group.

For T2D group, there was a significant difference in SBP after 30 min of recovery (rec30) compared to resting values. Moreover, ND group showed significant differences in post exercise SBP compared to resting values at rec30 and rec45. In addition, between-groups comparisons showed lower SBP values for ND group in relation to T2D group at rest, as well as during post-exercise recovery (rec15 and rec30) (Figure 2). Only the ND group presented PEH of mean arterial pressure (MAP) (rec45). On the other hand, only the T2D group presented PEH of diastolic blood pressure (DBP) (rec30 and rec45) (Figure 2).

## Discussion

The main finding of this study was that a single session of moderate exercise (90% LT) might decrease SBP in ND and T2D individuals during the post-exercise recovery period. However, the ND group presented a more pronounced PEH when compared to T2D individuals. Only the ND group showed PEH of MAP (rec45). This could be partially explained by a significant increase in post-exercise PKA (rec15) and the resultant increases in BK (rec45) and NOx concentrations (rec15). The higher levels of DesArg^9^-BK at rec45 for T2D in relation to ND group suggest a possible inhibitory effect of angiotensin converting enzyme on BK production. 

However, the T2D group was also responsive to a moderate intensity aerobic exercise, also presenting PEH of SBP at rec30 and of DBP at rec30 and rec45. This phenomenon may be related to the significant increase in post-exercise NOx concentration observed for both groups. However, compared to T2D group, the ND individuals presented a higher resting NOx concentration on the control session as well as after the end of exercise on EX session. Furthermore, PKA and BK were higher for ND in relation to T2D group at rec15 and rec45, respectively. Additionally, there were between-group differences in DesArg^9^-BK concentration at rec45, confirming the lower BK formation rate in the diabetic group.

Despite of higher NOx values for ND in relation to T2D group, the moderate intensity exercise session (90% of LT) used in the present study seemed to be effective for both groups in terms of promoting increases in NOx concentration that could be related to PEH.

 Thus, the initial hypothesis of the present study was confirmed, as post-exercise PKA, as well as the ability to release BK and NOx, seemed to be lower in patients with T2D, what may partially explain the attenuated response of post-exercise hypotension for this group when compared to ND individuals. The diabetic condition decreases the activity of the kallikrein-kinin system by reducing the synthesis of plasma prekallikrein [29] as a result of endothelial dysfunction [30]. On the other hand, studies have shown that a single exercise session can be effective to increase the activity of kallikrein-kinin system and consequently to increase NO bioavailability [31,32], BK concentration [33] and PKA [6,23,34], which are the main promoters of vasorelaxation and therefore of PEH.

However, the magnitude of PEH in patients with T2D was shown to be diminished, perhaps because this population commonly exhibit autonomic [35] and endothelial dysfunction [4,5]. These individuals also present an increased production of reactive oxygen species and lower levels of antioxidant markers [36], reducing the bioavailability of NO, depleting Tetrahydrobiopterin (BH4) – an important co-factor of nitric oxide synthase – and also increasing the damage to endothelial cells and smooth muscle [37]. As a consequence of the elevated oxidative stress, subjects with T2D also have a higher inflammation rate, which in turn inhibits NO release and impairs endothelial vasodilation [19]. Apart of that, these patients also show increased levels of vasoconstrictor substances [18,30]. In the present study, the exercise carried out at a moderate intensity was effective in increasing NOx release in type 2 diabetics. However, it appears that the ability of the endothelium of T2D individuals to respond to exercise is decreased, since NOx release, as well as BK concentration and PKA were lower, while DesArg^9^-BK values were higher when compared to ND group.

Some studies have shown a reduced activity of the kallikrein-kinin system in type 2 diabetics in response to exercise [6,33]. Thus, PEH seems to be dependent on the increased activity of this system [6,23]. In the present study, the lower PKA, BK concentration and NO production and the higher DesArg^9^-BK levels in subjects with T2D may have contributed to the lower magnitude of PEH observed for this group. Nevertheless, our results showed that even for these individuals with T2D, the exercise performed at a moderate intensity may still benefit blood pressure control as a result of increased NO levels in relation to pre-exercise resting values. 

Although the present study displays originality, especially with regards to the possible mechanisms involved in post-exercise hypotension, a potential limitation is the small sample size of patients. Nevertheless, the statistical power (1-β) *a priori* conferred to the analysis of comparison within and between groups (Split Plot ANOVA) for the interest variables (BK, kallikrein, DesArg^9^-BK, NOx and blood pressure) was 89% (Power = 0.89) considering an alpha of 5% and an effect size of f = 0.3, which is within the recommended range. Furthermore, it is important to highlight that endothelial function of the participants was not evaluated. However, as previously reported [4,5], individuals with T2D present low endothelial function compared to their non-diabetic peers.

 Interventions that increase NO bioavailability in individuals with T2D may be important to minimize cardiovascular dysfunction in this population [38] and thus needs further investigation to help determining the optimal dose of exercise for this population. Thus, the effects of an exercise performed at a different exercise intensity domain should be investigated in this population (additional dose-response studies).

## Conclusion

It can be concluded that a single exercise session performed at 90% LT (moderate-intensity) was effective in promoting PEH for both ND and T2D individuals, although the ND group showed a more pronounced and longer lasting PEH, which can be partially explained by the higher PKA, BK concentration and NO release compared to T2D group. Thus, it seems that the magnitude and duration of PEH in individuals with T2D also depends on the activity of the kallikrein-kinin system. Finally, additional studies on the role of exercise intensity on the investigated variables for individuals with T2D are necessary.
